# Short-Term Ultraviolet (UV)-A Light-Emitting Diode (LED) Radiation Improves Biomass and Bioactive Compounds of Kale

**DOI:** 10.3389/fpls.2019.01042

**Published:** 2019-08-20

**Authors:** Jin-Hui Lee, Myung-Min Oh, Ki-Ho Son

**Affiliations:** ^1^Division of Animal, Horticultural and Food Science, Chungbuk National University, Cheongju, South Korea; ^2^Brain Korea Center for Bio-Resource Development, Chungbuk National University, Cheongju, South Korea; ^3^Department of Horticultural Science, College of Life Science, Gyeongnam National University of Science and Technology, Jinju, South Korea

**Keywords:** antioxidant capacity, kale, phenolic compound, reactive oxygen species, transcript level, UV-A LEDs

## Abstract

The aim of this study was to determine the influence of two types of UV-A LEDs on the growth and accumulation of phytochemicals in kale (*Brassica oleracea* var. *acephala*). Fourteen-day-old kale seedlings were transferred to a growth chamber and cultivated for 3 weeks. The kale plants were subsequently subjected to two types of UV-A LEDs (370 and 385 nm) of 30 W/m^2^ for 5 days. Growth characteristics were all significantly increased in plants exposed to UV-A LEDs, especially at the 385 nm level, for which dry weight of shoots and roots were significantly increased by 2.22 and 2.5 times, respectively, at 5 days of treatment. Maximum quantum efficiency of photosystem II photochemistry (Fv/Fm ratio) began to decrease after 3 h of treatment compared to the control. The total phenolic content of plants exposed to the two types of UV-A LEDs increased by 25% at 370 nm and 42% at 385 nm at 5 days of treatment, and antioxidant capacity also increased. The two types of UV-A LEDs also induced increasing contents of caffeic acid, ferulic acid, and kaempferol. The reactive oxygen species (ROS) temporarily increased in plants exposed to the two types of UV-A LEDs after 3 h of treatment. Moreover, transcript levels of phenylalanine ammonia-lyase (PAL), chalcone synthase (CHS), and flavanone 3-hydroxylase (F3H) genes and PAL enzyme activity were higher in plants treated with UV-A LEDs. Our results suggested that short-term UV-A LEDs were effective in increasing growth and improving antioxidant phenolic compounds in kale, thereby representing a potentially effective strategy for enhancing the production of phytochemicals.

## Introduction

Extreme environmental factors, such as temperature (low and high), water availability (deficit and flooding), light (ultraviolet and high light), salinity, and nutrient level (deficit and excess) that inhibit plant growth can be defined as abiotic stresses. Plants that are subjected to a high level or chronic abiotic stresses during growth and development experience cell damage as a result of oxidative stress induced by excessive production of radicals or non-radical reactive oxygen species (ROS), which eventually may lead to necrosis. However, plants subjected to endurable levels of abiotic stress or temporary abiotic stress often adapt to the stressful environment using inherent defense mechanisms (Drew, 1998); that is, many plants are capable of adapting to environmental conditions unsuitable for growth and development by activating various enzymatic or non-enzymatic antioxidant biosynthetic pathways as defense mechanisms, which reduce ROS levels resulting from external abiotic stress. Non-enzymatic antioxidants generated in plants act in a similar manner in the human body when humans ingest plant-based foodstuff, and thus, they reduce the risk of chronic and cardiovascular diseases and cancer, as well as slowing the effects of aging, by eliminating excessive ROS. Because of these beneficial effects, bioactive compounds, including antioxidants of plant-based food items, are increasingly attracting both scientific and public attention. Recently, the application of mild abiotic stress during plant cultivation has been recognized as a potential strategy for enhancing the production of bioactive compounds in vegetables (Akula and Ravishankar, 2011; Lee and Oh, 2015).

Ultraviolet light (200–400 nm), an abiotic stressor, comprises up to 7% of the total sunlight reaching the Earth (Müller-Xing et al., 2014). The UV light is divided into UV-C (200–280 nm), UV-B (280–320 nm), and UV-A (320–400 nm) spectra, depending on wavelength. Irradiation with UV-C or UV-B, which are relatively high energy compared to UV-A, may cause fatal damage to plant biomolecules, proteins, and DNA, but UV-C is completely and UV-B mostly absorbed by the ozone layer of the atmosphere, with only a small amount of the latter (about 1.5%) present in the sunlight reaching the Earth’s surface. Approximately 98.7% of the UV radiation that reaches the Earth’s surface is in the form of UV-A (Yarosh and Smiles, 2009), which therefore represents the primary type of natural irradiation available to plants.

The degree of plant response to UV light generally depends on the amount of energy (dosage) and the range of the spectrum, but even excessive UV-A (above ambient) levels can cause necrosis of plant cells or disruption of the photosynthesis apparatus, which is similar to the damage caused by UV-B exposure (Bidel et al., 2015). It was recently reported, however, that exposure to low-levels of UV-A radiation promotes the production of antioxidant compounds and photosynthetic pigments in plants, and stimulates overall plant growth (Tsormpatsidis et al., 2008; Brazaitytė et al., 2010). Moreover, it was shown that supplemental UV-A light present in photosynthetically active radiation (PAR, 400–700 nm) enhanced chlorophyll and carotenoid concentrations, thereby promoting plant growth (Foyer et al., 1994; Ehling-Schultz et al., 1997). These results suggest that supplemental UV-A irradiation can improve the yield and/or quality of crops cultivated in greenhouses or otherwise grown under artificial light. However, the UV-A lamps most commonly used in previous research involving UV-A exposure encompass a wide range of UV-A wavelengths, including some visible light regions, making it difficult to identify the specific UV-A range or band responsible (Tsormpatsidis et al., 2008; Lee et al., 2014). Thus, a light source capable of intensive irradiation with a specific wavelength is required in order to explore the physiological responses of crop plants to the UV-A spectrum. Using UV-A LEDs that are energy efficient and have narrow peak wavelengths represent one potential approach, but little research has been carried out on the physiological responses of plants to such light sources.

We hypothesized that short-term UV-A radiation would increase the content of phenolic compounds without growth inhibition or with minimum inhibition. Our objective here was to determine the effects of irradiation with different types of UV-A LEDs on the growth and production of antioxidant phenolic compounds in kale, which are known to be rich in bioactive compounds, immediately before harvest, as a means of exploring the feasibility of using UV-A LED irradiation to improve kale quality without affecting plant growth or yield.

## Material and Methods

### Plant Materials and Growth Conditions

Kale seeds (*Brassica oleracea* var. *acephala*) were sown on seed growth packs (Seed Pack, Useem Instruments, Inc., Suwon, Korea) and germinated in an environmentally controlled closed plant production system [air temperature 20°C; relative humidity 60%; CO_2_ 400 μmol mol^−1^; light period 12 h; photosynthetic photon flux density (PPFD) 136 ± 5 μmol m^−2^ s^−1^ irradiated by fluorescent lamp] for 2 weeks. The 2-week-old seedlings (total of 144 seedlings) were then transferred to a deep-flow technique (DFT) system and cultivated for a further 3 weeks. Half-strength Hoagland & Arnon nutrient solution was used for mineral supply resources, and electrical conductivity (EC) and pH levels were corrected to 1.2 dS m^−1^ and 6.0, respectively, with a digital multi-parameter (Multi 3430; WTW, Weilheim, Germany) once every 3 days.

### UV-A Treatments

**Figure 1:**
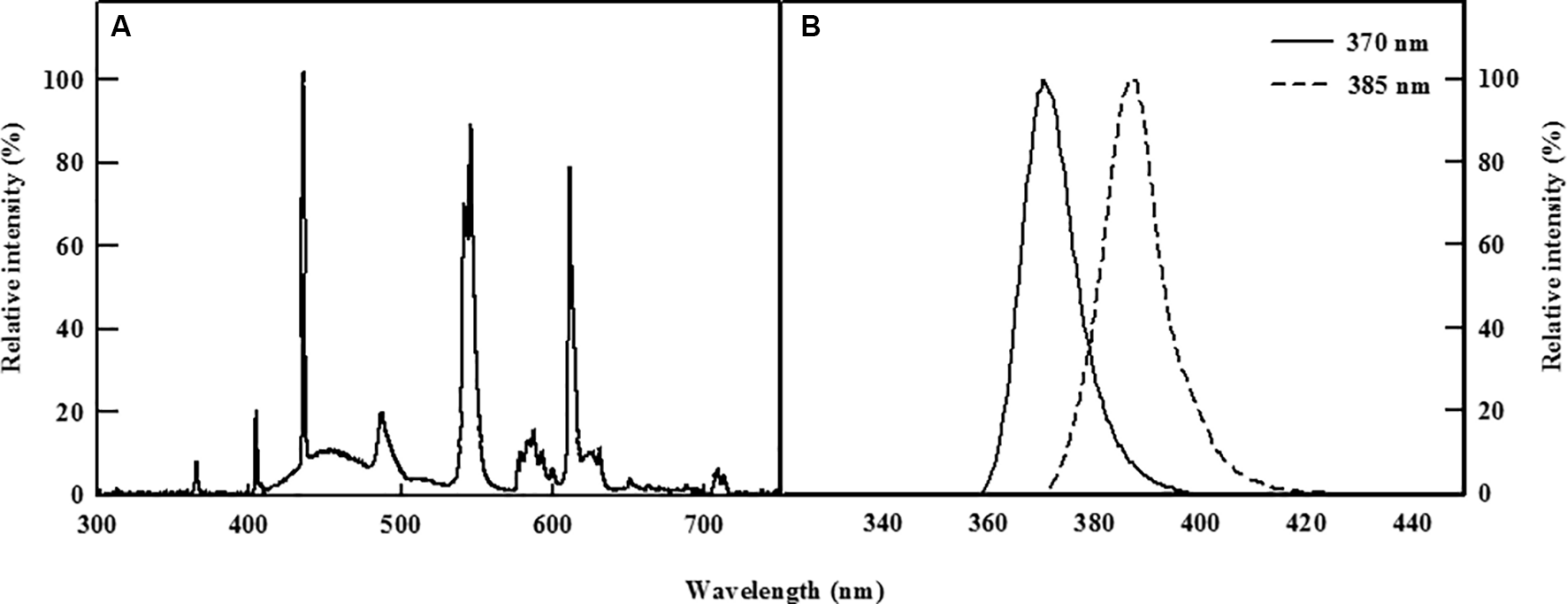
Relative spectral distribution of the fluorescent lamp **(A)** and the two types of UV-A LEDs with 370 or 385 nm peak wavelength used **(B)**.

Two types of commercially available UV-A LEDs (370 and 385 nm, LG Innotek, Seoul, South Korea, **Figure 1**) were used as supplemental light sources to irradiate the kale cultivated in the DFT system continuously for 5 days. Light energy levels at the level of the plant canopy were set to 30 W/m^2^ for both types of UV LEDs using Jaz spectrometer (Ocean Optics, Dunedin, FL, USA), whereas control plants were grown for 5 days under a normal existing fluorescent lamp.

### Biomass Characteristics

In order to confirm the change of kale growth by UV-A LED treatment, kale biomass and leaf area were measured immediately before UV treatment and after 5 days of UV treatment. Fresh weights of shoots and roots were measured using an electronic scale (SI-234; Denver Instruments, Denver, CO, USA), and leaf area was measured using an area meter (LI-3100; LI-Cor, Lincoln, NE, USA). After measuring the fresh weight, shoots and roots were oven-dried separately (VS-1202D3; Vision Scientific, Daejeon, Korea) at 70°C, following which dry weights of shoots and roots were determined.

### Photosynthetic Rate and Chlorophyll Fluorescence

To assess changes in the photosynthetic rate of plants subjected to UV-A irradiation, CO_2_ concentrations in the fourth fully expanded leaf were measured using a portable photosynthesis system (LI-6400; Li-Cor, Lincoln, NE, USA) at 4 days of UV-A LED treatment. Airflow rate, CO_2_ concentration, and the temperature inside the leaf cuvette were maintained at 350 μmol s^−1^, 400 μmol mol^−1^, and 20°C, respectively.

Maximum quantum efficiency of photosystem II photochemistry (Fv/Fm) was measured in the third leaf from the top of the shoot immediately before UV exposure and at first 3 h and then 12-h intervals during UV treatment using a chlorophyll fluorescence meter (PAM 2000; Heinz Walz GmbH, Effeltrich, Germany) to determine the stress level of plants receiving UV-A LED irradiation. Leaves were allowed to adjust to the dark for 30 min prior to the measurement, and maximum fluorescence (Fm) and minimum fluorescence (Fo) levels of the leaves were measured. Saturating light pulse and intensity was 20 kHz and 1,100 μmol m^−2^ s^−1^ PPFD, respectively. Ratios of Fv/Fm were calculated using the equation Fv/Fm = (Fm−Fo)/Fm (Maxwell and Johnson, 2000).

### Total Phenolic Content and Antioxidant Capacity

Total phenolic content and antioxidant capacity were evaluated in order to examine how beneficial bioactive compounds were affected by UV-A irradiation immediately before treatment and at 3 h and 1, 2, 3, 4, and 5 days during UV treatment over the entire experimental period. The kale fresh leaf was used for the analysis. Approximately 200 mg of fresh leaf sample was collected and stored in a deep freezer (−70°C) until analysis. Total phenolic content was analyzed using the Folin-Ciocalteu method (Ainsworth and Gillespie, 2007), with minor modifications. Frozen samples were transferred to a mortar and then ground with liquid nitrogen. Acetone (80%) was used for extraction, and the solution was incubated at 4°C for 12 h. The extraction solution was centrifuged at 3,000 × *g* for 2 min, with the supernatant used in the analysis. The remaining analytical procedures followed those described by Lee and Oh (2015), and units were expressed as gallic acid equivalent (mg) per fresh weight (g) (GAE mg/g FW). Antioxidant capacity was determined *via* a slightly modified method using 2,2′-azino-bis (3-ethylbenzothiazoline-6-sulfonic acid) (ABTS; Sigma-Aldrich, St. Louis, MO, USA) (Miller and Rice-Evans, 1996). The samples were extracted in the same manner as those used in the analysis of total phenolic content and incubated at −20°C for 12 h, then centrifuged at 3,000 × *g* for 2 min, with the resulting supernatant used for the analysis. The remaining analytical procedures followed those described by Lee and Oh (2015). A standard curve was established using 6-hydroxy-2,5,7,8-tetramethylchroman-2-carboxylic acid (Trolox; Sigma-Aldrich, St. Louis, MO, USA), and the units were expressed as Trolox-equivalent antioxidant capacity (mM) per fresh weight (g) (TEAC mM/g FW).

### Individual Phenolic Compounds

To analyze changes in the contents of individual phenolic compounds, kale leaf was collected immediately before and 3 h and 1, 2, 3, 4, and 5 days after initiation of UV-A exposure. The samples (0.5 g) were frozen in liquid nitrogen and stored at −70°C until analysis, at which time they were ground in liquid nitrogen and extracted using acetonitrile and 0.5% hydrochloride acid solution. Extraction was performed as earlier described by Lin and Harnly (2009) with some modifications. The extracts of kale were subjected to acid hydrolysis to obtain the phenolic compounds aglycones for characterization. The extraction solution was acid-hydrolyzed for 2 h in a water bath at 85°C, after which the solution was sonicated (Sk5210HP; Hangzhou Nade Scientific Instruments, Zhejiang, China) for 90 min. Following centrifugation at 3,000 × *g* for 20 min, the supernatant was filtered through a syringe filter with a pore size of 0.22 μm (WKSPU0230; Woongki, Seoul, South Korea). Analysis of each of the phenolic compounds was performed with a high-performance liquid chromatography (HPLC) system (YL9100; Younglin, Anyang, Korea). An Agilent Eclipse Plus-C18 column (4.6 mm × 150 mm, 5µm; Agilent Technology, Santa Clara, CA, USA) equipped with a guard column was used. The column was operated at a temperature of 30°C and an injection volume of 10 µl. The mobile phase consisted of 100% acetonitrile (eluent A) and 0.5% acetic acid in water (eluent B). The gradient program consisted of 0–10 min: 10% A; 10–30 min: 20% A; 30–40 min: 30% A; 40–50 min: 40% A; 50–60 min: 80% A; 60–61 min: 0% A; and 61–70 min: 0% A (flow rate of 0.8 ml min^−1^). Absorbance was recorded at 320 nm. Caffeic acid, ferulic acid, and kaempferol (Sigma-Aldrich, St. Louis, MO, USA) were used as standards. Contents of the individual phenolic compounds were expressed as mg per 100 g fresh weight (mg/100 g FW).

### Reactive Oxygen Species (H_2_O_2_ and O_2_^·−^)

Hydrogen peroxide (H_2_O_2_) content was measured following the method described by Aroca et al. (2003). Approximately 0.5 g of fresh leaf tissue was collected (before and 3 h and 1, 2, 3, 4, and 5 days of UV-A treatment) and stored in a deep freezer (DF8524; IlShinBioBase, Dongducheon, Korea) at −70°C until analysis. Kale samples were ground in a mortar with liquid nitrogen and homogenized with 5 ml of 5% (w/v) trichloroacetic acid (TCA) containing 0.1 g of activated charcoal and 1% (w/v) polyvinylpolypyrrolidone (PVPP). The homogenized sample was centrifuged at 18,000 × *g* for 10 min, and the supernatant was filtered through a 0.22 µm syringe filter. Following the addition of 1.2 ml of 100 mM potassium phosphate buffer (pH = 8.4) and 0.6 ml of colorimetric reagent to 130 μl of the supernatant, the solution was incubated at 45°C for 1 h and absorbance was recorded at 508 nm using a spectrophotometer (UV-1800, Shimadzu, Kyoto, Japan). The colorimetric reagent was made by mixing 0.6 mM potassium titanium oxalate and 0.6 mM 4-2 (2-pyridylazo) resorcinol (disodium salt) as 1:1 (v/v). Blanks were constructed with 5% TCA instead of the extracted solution, and units were expressed as H_2_O_2_ (nmol) per fresh weight (g) (nmol g^−1^ FW).

Superoxide radical (O_2_^·−^) content was measured following the method described by Wang and Luo (1990). Approximately 0.5 g of frozen sample was made into powder by grinding with liquid nitrogen and subsequently mixed with 5 ml of 50 mM potassium phosphate buffer (pH = 7.8). After centrifuging at 5,000 × *g* for 15 min, 1 ml of the supernatant was mixed with 0.9 ml of 50 mM potassium phosphate buffer (pH = 7.8) and 0.1 ml of 10 mM hydroxylamine hydrochloride. The mixed solution was incubated at 25°C for 30 min, and subsequently, 1 ml of 17 mM 3-aminobenzenesulfonic acid and 1 ml of 7 mM 1-naphthylamine were added to 1 ml of the incubated solution. The solution was once again incubated at 25°C for an additional 20 min, following which absorbance was measured at 530 nm. Units were expressed as mmol of O_2_^·−^ per hour per gram of fresh weight (mmol O_2_^·−^ h ^−1^ g^−1^ FW).

### Phenylalanine Ammonia-Lyase (PAL) Enzyme Activity

Phenylalanine ammonia-lyase (PAL) activity was analyzed following the method of Boo et al. (2011), with minor modification. The fresh leaf segment (∼0.5 g) was used for the analysis. Samples were collected immediately prior to UV exposure and at first 3 h and then 1-day intervals over the course of 5 days of UV-A treatment and stored in a deep freezer (DF8524; IlShinBioBase, Dongducheon, Korea) at −70°C until analysis. We used the method described by Lee and Oh (2015) for the analysis. The standard curve was constructed by trans-cinnamic acid (Sigma-Aldrich, St. Louis, MO, USA). Units were expressed as mM of trans-cinnamic acid per hour per gram of fresh weight (mM trans-cinnamic acid h ^−1^ g^−1 ^FW).

### Transcriptional Levels of PAL, CHS, and F3H Genes

To compare the transcriptional levels of the PAL, chalcone synthase (CHS), and flavanone 3-hydroxylase (F3H) genes, which are essential for biosynthesis of phenolic compounds in kale leaves, leaf segment was collected (∼0.2 g) (before and 3 h and 1, 2, 3, 4, and 5 days of UV-A treatment) and stored at −70°C in a deep freezer until analysis. Total RNA was extracted using an RNeasy Plant Mini Kit (74904; QIAGEN, Hilden, Germany), and the concentration was checked with a NanoDrop 1000 Spectrophotometer (Thermo Scientific, Wilmington, USA), whereas cDNA synthesis was performed with a QuantiTect Reverse Transcription Kit (QIAGEN, Hilden, Germany). Quantitative real-time PCR was performed using a 2 × QuantiMix SYBR Kit (PhileKorea, Daejeon, Korea) for the PAL gene, and a 2 × MakTaq ReMix (T121M, QIAGEN, Hilden, Germany) for the CHS and F3H genes. Products of PAL, CHS, and F3H were amplified with a Rotor-gene 6000 (Corbett Life Science, Sydney, Australia). The oligonucleotide primers used in the experiments were constructed based on information obtained from the GenBank database (**Table 1**). The PCR conditions for the PAL gene were as follows: 95°C for 5 min for the pre-denaturing stage and in the subsequent 33 cycles, consisting of denaturing (95°C for 30 s), annealing (60°C for 120 s), and extension (72°C for 90 s). The PCR conditions for the CHS and F3H genes were as follows: 95°C for 2 min for the pre-denaturing stage and in the subsequent 40 cycles, consisting of denaturing (95°C for 5 s), annealing (60°C for 20 s), and extension (72°C for 20 s). The degree of relative gene expression (ΔΔCT) was calculated using Rotor-gene 1.7 software (QIAGEN, Hilden, Germany).

**Table 1:** List of oligonucleotide primers used for quantitative-real-time PCR.

Gene name^z^	Forward primer sequence	Reverse primer sequence	PCR product (bp)
*BoActin*	GAACTACGAGTTGCCCGACG	GCAGCTTCCATTCCCACGAA	20/20
*BoPAL*	CTCGACCCTTGGAAACGGTG	CCGTTCTTGGTTCTCCGGTG	20/20
*BoCHS*	CCCTCTGACACCCACCTTGA	GCGGCAGACACCATCTCAAA	20/20
*BoF3H*	CGGCGTGGATGTGAAAGGAA	CAGCTTCTCCGGCGTAACTC	20/20

^z^GenBank accession numbers for Actin, PAL, CHS, and F3H are KF218591.1, FJ849059.1, EF531094.1, and EF531096.1, respectively.

### Statistical Analysis

Statistical analyses were performed with SAS (SAS 9.2; SAS Institute, Cary, NC, USA). Each parameter included four biological replicates except for photosynthetic rate and Fv/Fm, which had five biological replicates and subjected to analysis of variance (ANOVA). The experiment was repeated twice to confirm reproducibility. Tukey’s range test (HSD) was used to compare the means between treatments, with statistical significance expressed at *p* < 0.05.

## Results

### Growth Characteristics

Fresh and dry weights of shoots and roots, leaf area, and specific leaf weight were significantly higher in kale irradiated with the two types of UV-A LEDs for 5 days (**Table 2**). Shoot fresh weight exposed to 370 nm and 385 nm wavelengths increased by 1.32 and 1.58 times, and shoot dry weight by 1.57 and 1.95 times, respectively, compared to control plants. In addition, fresh and dry weights of roots increased in plants subjected to both UV-A treatments and significantly increased (by 2.22 and 2.5 times) in plants exposed to 385 nm wavelengths compared to control plants, respectively. In addition, leaf area in plants treated with UV-A LEDs at wavelengths of 385 nm was 1.42 times higher than the leaf area of control plants, and specific leaf weight (an indication of leaf thickness) was also significantly higher, by 1.15 and 1.43 times, in plants exposed to UV-A wavelengths of 370 and 385 nm, respectively, compared to controls.

**Table 2:** Growth characteristics of kale immediately prior to UV treatment and 5 days of UV treatments (n = 4).

Time	Treatment	Shoot		Root		Leaf area (cm^2^)	Specific leaf weight (mg cm^−2^)
		Fresh weight (g)	Dry weight (g)	Fresh weight (g)	Dry weight (g)		
Before treatment Z		4.18 ± 0.20^y^	0.31 ± 0.01	0.52 ± 0.05	0.03 ± 0.01	107.02 ± 5.31	0.0029
5 days of treatment	Control	7.19 ± 0.36b^x^	0.52 ± 0.03b	0.70 ± 0.07b	0.05 ± 0.01c	179.34 ± 6.74b	0.0029b
	370 nm	9.49 ± 0.56ab	0.83 ± 0.04a	1.09 ± 0.06b	0.09 ± 0.01b	207.52 ± 11.38b	0.0040a
	385 nm	11.39 ± 0.90a	1.03 ± 0.09a	1.57 ± 0.17a	0.12 ± 0.01a	255.97 ± 14.25a	0.0040a
	Significance	**	***	**	***	***	**

^z^UV non-treated plant

^y^Data are presented as mean ± SE.

^x^Mean separation within columns by Tukey’s range test (HSD).

**, ***Significant at p < 0.01 and p < 0.001, respectively.

### Photosynthetic Rate and Chlorophyll Fluorescence

**Figure 2:**
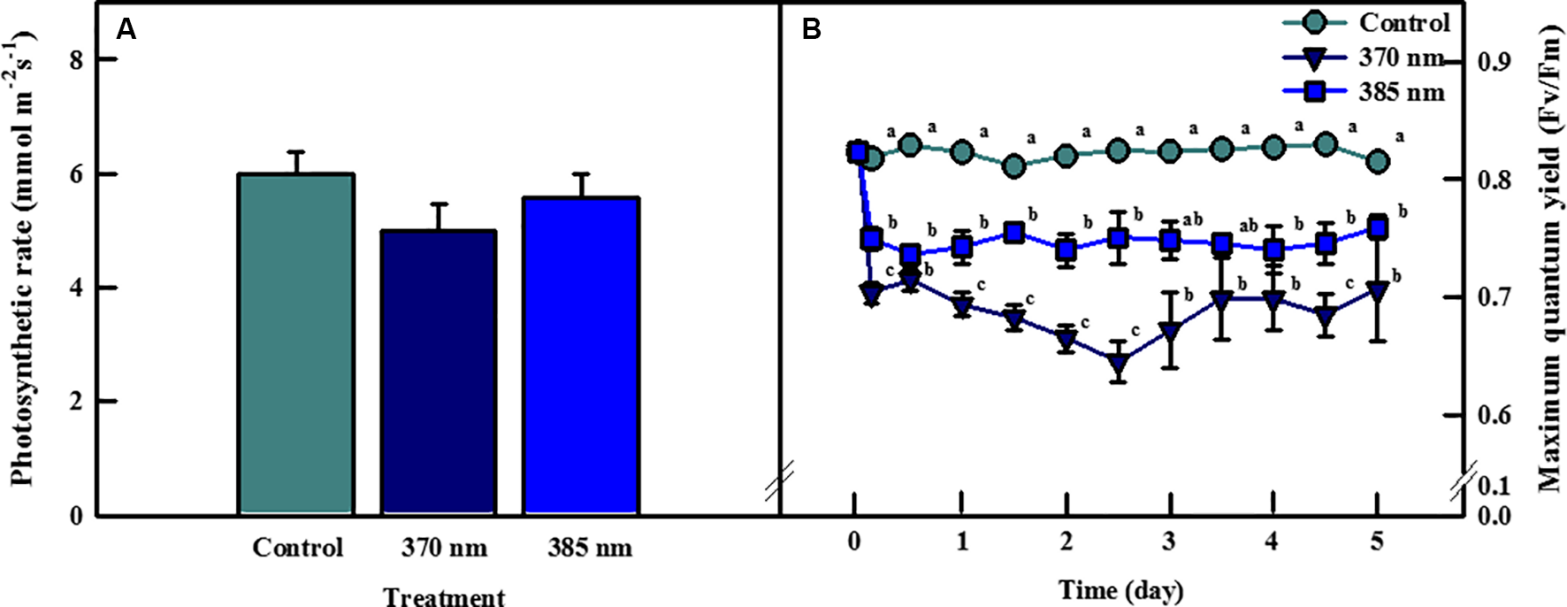
Photosynthetic rate of kale subjected to two types of UV-LED lights (370 and 385 nm) at 4 days **(A)** and maximum quantum efficiency of photosystem II (Fv/Fm) of kale subjected to two types of UV-LED lights (370 and 385 nm) for 5 days **(B)**. The vertical bars indicate standard errors (n = 5). Different letters indicate significant differences, as assessed by ANOVA (*p* < 0.05).

Photosynthetic rates of kale leaves irradiated by UV-A LEDs for 4 days are shown in **Figure 2A**. The highest photosynthetic rate was observed in the control plants, followed by plants exposed to UV-A at wavelengths of 385 and 370 nm, respectively, but none of these differences were statistically significant. However, significant differences were detected between control plants and those exposed to the two types of UV-A LEDs in terms of maximum quantum efficiency of photosystem II photochemistry (Fv/Fm), a non-destructive stress indicator (**Figure 2B**). The Fv/Fm ratio rapidly decreased over the initial 3 h of UV-A LED irradiation (by 0.70 at 370 nm and 0.75 at 385 nm) and remained below 0.8 over the entire period of UV-A LED exposure. The Fv/Fm ratio in plants exposed to the 385 nm wavelength ranged from 0.73 to 0.76, whereas in plants exposed to the 370 nm treatment the Fv/Fm ratio was 0.04–0.1 lower than the 385 nm plants at 1–4 days of UV-A irradiation.

### Total Phenolic Content and Antioxidant Capacity

**Figure 3:**
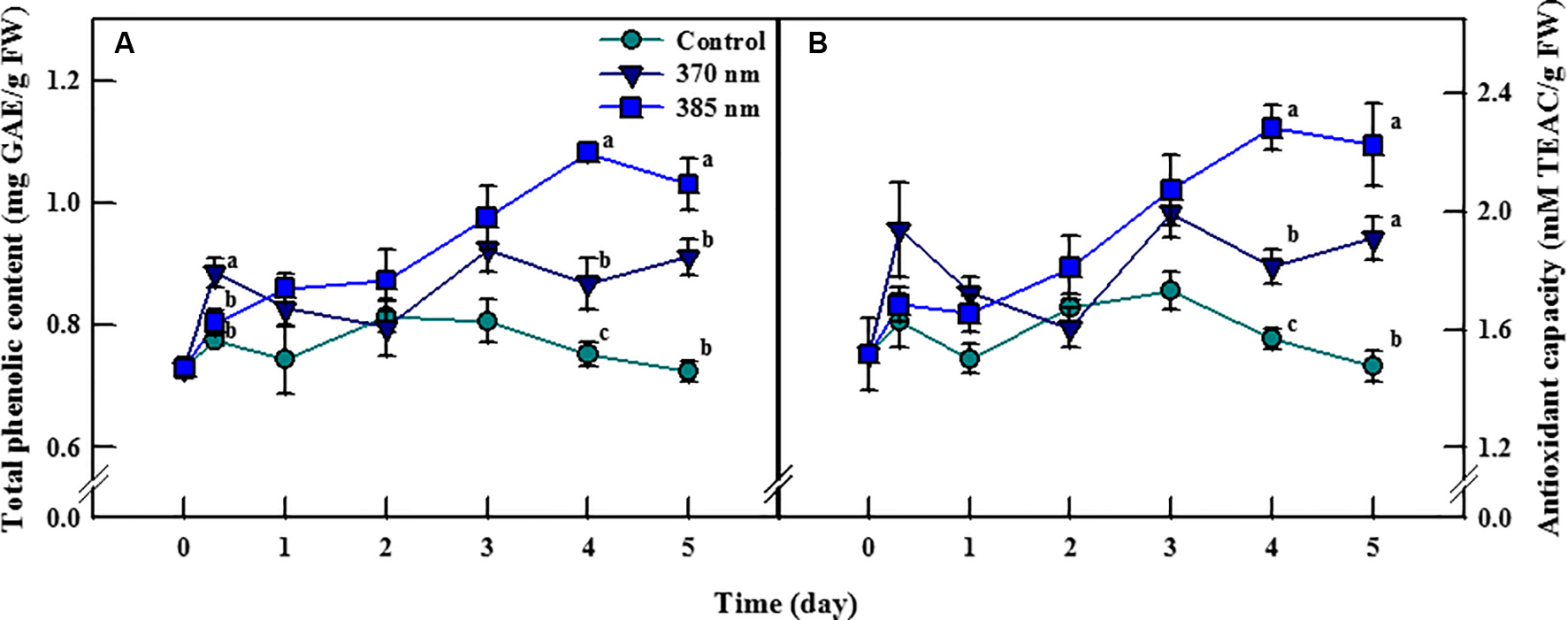
Total phenolic content **(A)** and antioxidant capacity **(B)** of kale subjected to two types of UV-A LED lights for 5 days. The vertical bars indicate standard errors (n = 4). Different letters indicate significant differences, as assessed by ANOVA (*p* < 0.05).

Total phenolic content and antioxidant capacity of kale leaves differed according to time, and the peak wavelength of UV-A LED irradiation (**Figure 3**). Total phenolic content in plants exposed to UV-A 370 nm significantly increased, by 14%, compared to the control following 3 h of UV-A irradiation, then decreased over the next 2 days of treatments, but significantly increased thereafter, by 15% and 26% after 4 and 5 days of treatment, respectively. Total phenolic content also increased in plants treated with UV-A 385 nm for the first 1–3 days, and significantly so by days 4 and 5, by 44% and 42% compared to control plants, respectively. Antioxidant capacity exhibited a similar trend: at 4 and 5 days of UV-A LEDs treatment, plants exposed to UV-A 370 nm experienced a significant increase in antioxidant capacity of 15% and 29%, respectively, and that of plants exposed to UV-A 385 nm by 45% and 51%, respectively, compared to control plants.

### Individual Phenolic Compounds

**Figure 4:**
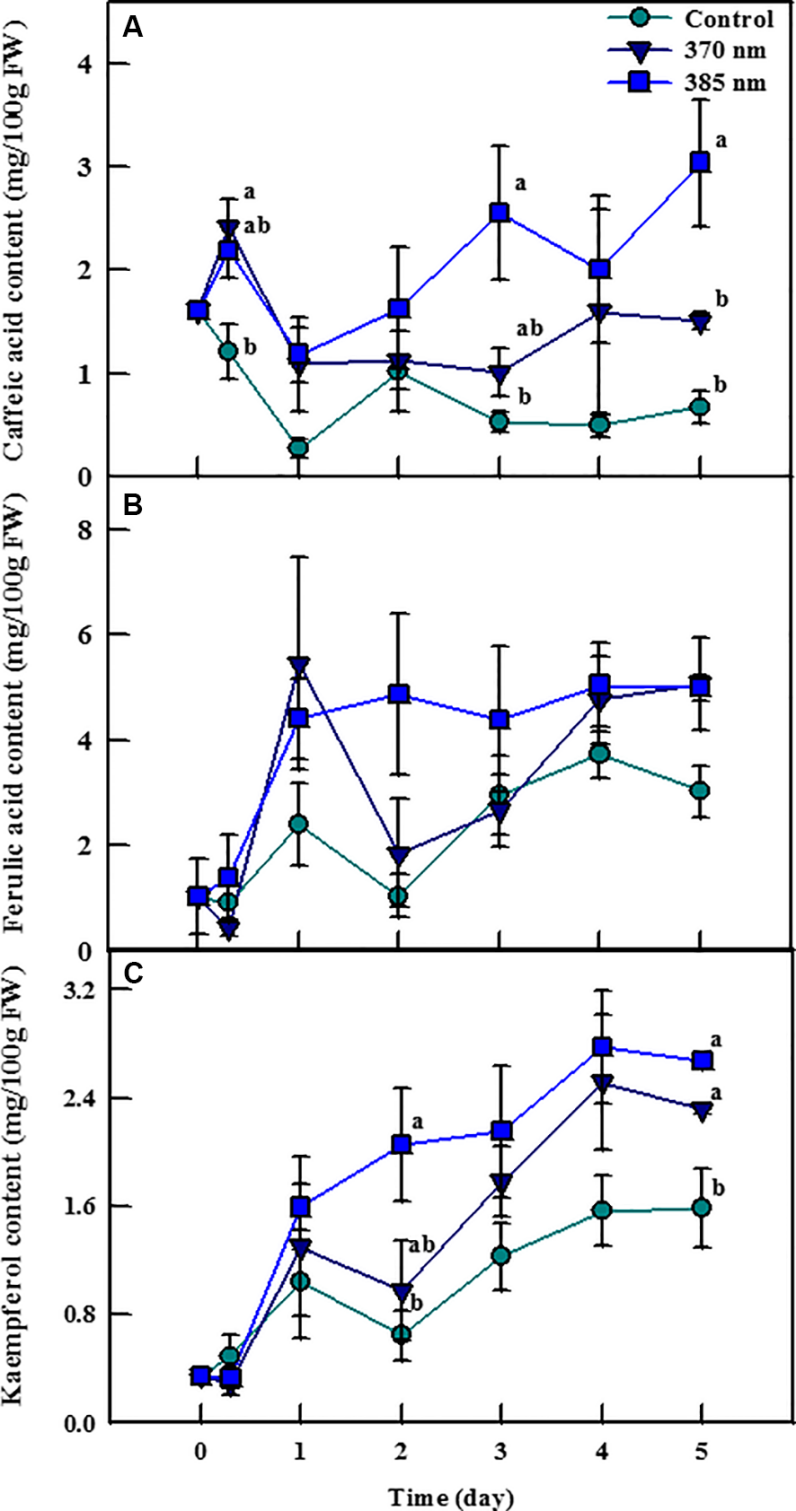
Individual phenolic compounds [caffeic acid; **(A)** ferulic acid; **(B)** and kaempferol; **(C)**] of kale subjected to two types of UV-A LED lights for 5 days. The vertical bars indicate standard errors (n = 4). Different letters indicate significant differences, as assessed by ANOVA (*p* < 0.05).

Irradiation with UV-A LEDs was effective in enhancing the contents of the phenolic compounds caffeic acid, ferulic acid, and kaempferol (**Figure 4**). Caffeic acid contents were 2 and 1.82 times higher in plants exposed to UV-A 370 nm and UV-A 385 nm, respectively, than in control plants within 3 h of treatment, and remained significantly higher thereafter. Most notably, plants treated with UV-A 385 nm had 4.86 and 4.55 times more caffeic acid than control plants after 3 and 5 days of treatment, respectively. Ferulic acid contents were also higher in plants exposed to the two UV-A LEDs treatments, but these differences were not statistically significant. Finally, kaempferol contents exhibited a pattern similar to that of caffeic acid; UV-A 385 nm plants had 3.18 times as much kaempferol as did control plants after 2 days of treatment, a statistically significant difference, whereas kaempferol contents were 1.46 and 1.68 times higher in plants treated with UV-A 370 nm and UV-A 385 nm compared to control plants after 5 days of treatment, respectively, which were also statistically significant.

### Reactive Oxygen Species (H_2_O_2_ and O_2_^•-^)

**Figure 5:**
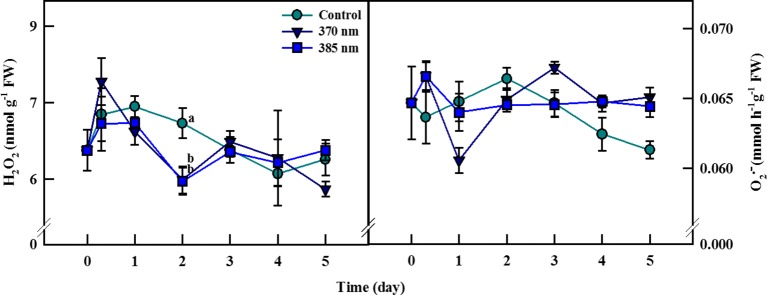
Hydrogen peroxide **(A)** and superoxide radical **(B)** of kale subjected to two types of UV-A LED lights for 5 days. The vertical bars indicate standard errors (n = 4). Different letters indicate significant differences, as assessed by ANOVA (*p* < 0.05).

The dynamics of ROS contents in plants under UV-A irradiation differed from that in control plants (**Figure 5**). Hydrogen peroxide contents were 8% higher in the 370 nm treated plants than in control plants at 3 h of UV-A treatment, after which they decreased continuously until 2 days of treatment, at which point H_2_O_2_ contents were 8% lower than in the controls. Hydrogen peroxide content then recovered to levels similar to control plants after 3 days of treatment, but then declined once more at 5 days of treatment. For plants exposed to UV-A 385 nm, H_2_O_2_ levels were slightly lower than in control plants after 3 h of treatment and 19% lower by 2 days of treatment. At 5 days of treatment, however, H_2_O_2_ contents were more or less the same in both treated and control plants.

Superoxide contents increased by 5% at 3 h but then decreased by 7% after 1 day of treatment in UV-A 370 nm plants compared to controls, although contents remained higher in the treated plants after 3 days of treatment. For kale receiving UV-A 385 nm, superoxide content increased to the same level as that for plants treated with UV-A 370 nm at 3 h but fell to control levels at 1 day of treatment. In this case, however, superoxide contents began to increase after 4 days of treatment and were approximately 5% higher than in control plants by 5 days.

### PAL Enzyme Activity

**Figure 6:**
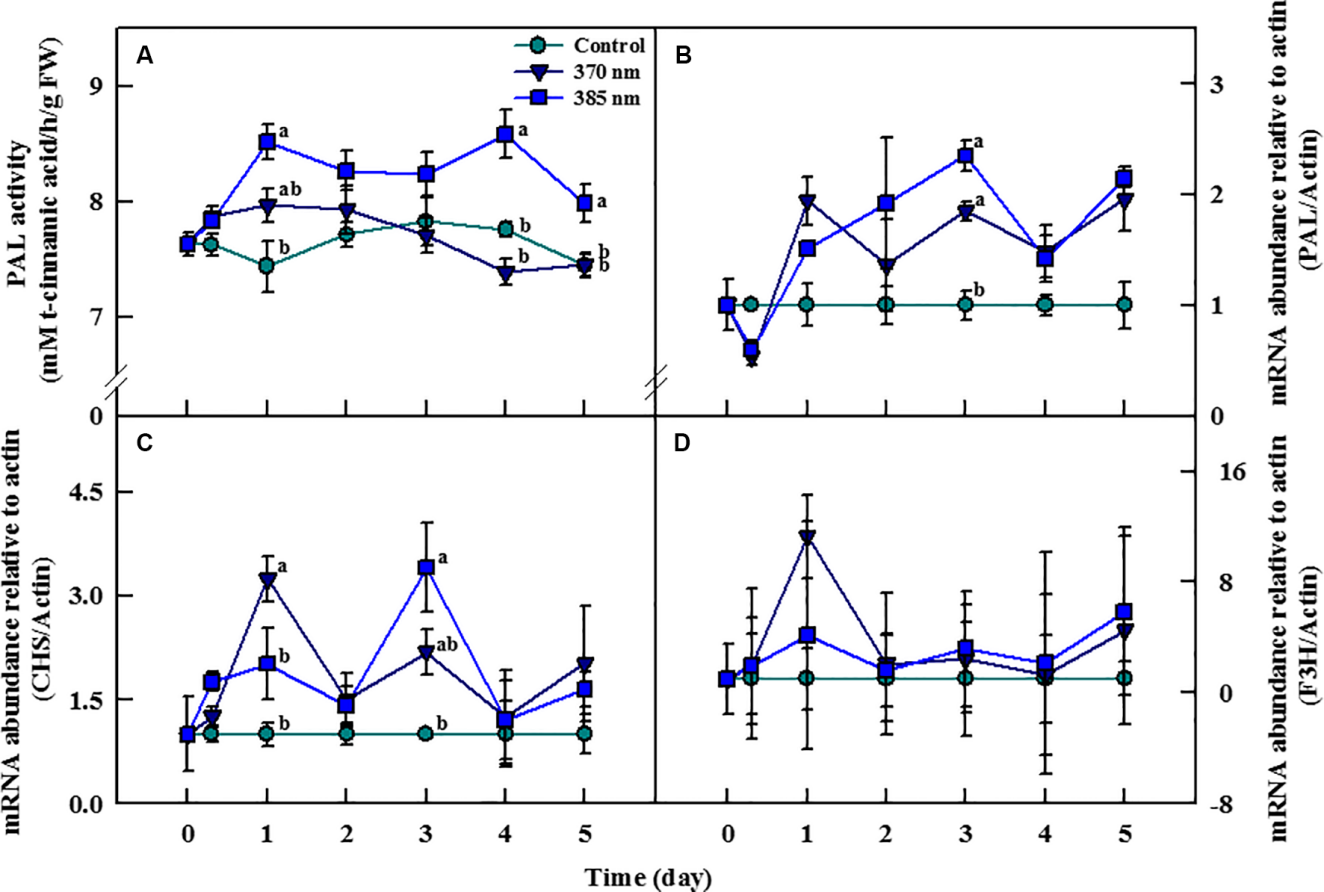
Phenylalanine ammonia-lyase (PAL) activity **(A)** and transcript levels of PAL **(B)**, chalcone synthase (CHS) **(C)**, and flavonoid 3-hydroxylase (F3H) genes **(D)** of kale subjected to two types of UV-A LED lights for 5 days. Gene expression levels were normalized to that of actin gene, a housekeeping gene. The vertical bars indicate standard errors (n = 4). Different letters indicate significant differences, as assessed by ANOVA (*p* < 0.05).

The activity of the PAL enzyme—a gateway enzyme in the biosynthesis pathway of secondary metabolites (phenolic compounds)—was significantly higher in plants treated with UV-A 385 nm at 1 day of treatment, with activity levels 44% higher than in the control plants (**Figure 6A**). PAL enzyme activity slightly decreased until 3 days of treatment in UV-A irradiated kale, but remained significantly higher, by 11% and 7%, than in control plants at 4 and 5 days of treatment, respectively. For plants receiving UV-A 370 nm, PAL activity increased at 1 day of treatment, but thereafter, there no significant differences in PAL activity were detected between the treated plants and the controls.

### Transcript Levels of the PAL, CHS, and F3H Genes

Transcript levels of the PAL, CHS, and F3H genes changed considerably with the time of exposure to UV-A irradiation (**Figures 6B–D**). Transcript levels of the PAL gene were lower at both 370 and 385 nm treated plants than in controls at 3 h of treatment, but then increased beginning 1 day after the initiation of the treatments. In plants treated with UV-A 370 nm, PAL transcript levels were higher than in plants treated with UV-A 385 nm but increased more rapidly in the UV-A 385 nm plants than in the UV-A 370 nm plants after 1 day of treatment. Transcription of the PAL gene increased by 1.8 and 2.3 times in the UV-A 370 and UV-A 385 plants compared to controls at 3 days of treatment (**Figure 6B**). Transcript levels of the CHS gene also drastically increased in both UV-A 370 and UV-A 385 nm plants at 1 day of treatment, by 3.2 and 2 times compared to the controls, respectively. However, the CHS gene was deactivated in plants of both treatments after 2 days, then reactivated at 3 days; overall, CHS gene transcription was higher in plants exposed to UV-A 385 nm (3.4 times compared to controls) than in plants exposed to UV-A 370 nm (2.1 times), a pattern similar to that for the PAL gene (**Figure 6C**). Finally, although transcript levels of the F3H gene were higher in UV-A treated kale than in control plants at 1, 3, and 5 days of treatment, these differences were not statistically significant (**Figure 6D**).

## Discussion

### Biomass

UV-A light effect on plant growth and development is known to be regulated by several factors such as environmental condition, dose of UV energy, plant species, and genotype (Cooley et al., 2001). However, exposure to UV light is generally believed to have deleterious effects on plant growth; for example, UV-A irradiation of 69.33 µmol m^−2^ s^−1^ and UV-B of 5.10 µmol m^−2^ s^−1^ were shown to inhibit the growth of *Amaranthus tricolor* (Kataria et al., 2013), and three cultivars of *Triticum sativum* subjected to UV-A of 63.5 W/m^2^ and UV-B of 2.7 W/m^2^ also exhibited slower growth rates (Häder, 1996). In our study, however, short-term exposure to two types of UV-A LEDs with 370 nm or 385 nm peak wavelength induced significant increases in several growth parameters of kale, a result consistent with those reported by Brazaitytė et al. (2015), in which growth-related parameters such as plant height, leaf area, and fresh weight were enhanced in basil, beet, and pak choi microgreens following exposure to UV-A LEDs with peak wavelengths of 390 and 402 nm. In addition, lettuce growth improved when plants were irradiated with UV-A in the bandwidth of 370–400 nm and the remaining UV-A range and UV-B were excluded *via* UV film under sunlight (Tsormpatsidis et al., 2008). These results suggest that some UV-A LED wavelengths are used in photosynthesis in addition to the visible light regions (> 400 nm) (**Figure 1**), and/or that UV-A wavelengths close to 400 nm might also be light sources available for photosynthesis. This is supported here by the comparative superiority of UV-A LED 385 nm than 370 nm in inducing positive responses in growth parameters in kale (**Table 1**). Additional evidence can be seen in the results of Brazaitytė et al. (2015), in which basil and pak choi subjected to several types of UV-A LEDs of 6.2 µmol m^−2^ s^−1^ (peak wavelengths: 366, 390, and 402 nm) exhibited improved plant height, hypocotyl length, fresh weight, and chlorophyll index at longer peak wavelengths. Verdaguer et al. (2017) noted that direct absorption of UV-A light energy by chlorophyll and carotenoids enhanced photosynthetic activity when UV-A was supplemented under low PAR conditions, implying that UV-A LED irradiation could be used as an additional source of light energy available for use in photosynthesis when light intensities fail to reach the light saturation point. Blue–green fluorescence excited from UV-A light absorbed by secondary metabolites such as phenolic compounds in leaf cells can be used indirectly as photosynthetic energy (Mantha et al., 2001; Johnson and Day, 2002). In our study, irradiation with UV-A was at a lower intensity (136 µmol m^−2^ s^−1^ of PPFD) than the light saturation point (> 400 µmol m^−2^ s^−1^) of kale, yet the photosynthetic rates of plants exposed to UV-A 370 nm and UV-A 385 nm were slightly lower than that of control plants at 4 days of treatment, although these differences were not statistically significant (**Figure 2A**). The inconsistency of the results for the growth and photosynthetic rates can be explained by the fact that in our experiment plants were continuously irradiated with UV-A LED for 5 days regardless of the period of visible light, and therefore, plants receiving UV-A were exposed to higher levels of the daily light integral (DLI) required for growth promotion than were control plants, which may have contributed to the improvements observed in their growth-related parameters (Torres and Lopez, 2011). In addition, increases in stomatal conductance, leaf soluble proteins, and nitrogen uptake by UV-A light may be another reason of positive effect on the photosynthesis (Tezuka et al., 1994; Mantha et al., 2001). Moreover, photoreceptors related to UV-A light are involved in long-distance signal transmission to regulate root growth (Zhang et al., 2014), resulting in the accumulation of root biomass as well as shoot biomass.

### Chlorophyll Fluorescence

Most of the light energy absorbed by chlorophylls is used for photosynthesis, and the rest is re-irradiated in the form of heat and fluorescence. Minimum fluorescence (Fo) indicates the smallest amount of fluorescence generated by chlorophyll molecules in the PS II antenna before the light energy moves to the PS II reaction center, whereas and maximum fluorescence (Fm) indicates the largest amount of fluorescence emission of a dark-adapted plant exposed to a short pulse of a strong light, leading to a transient reduction of quinone acceptor. Thus, the Fv/Fm (= Fm-Fo)/Fm) ratio quantifies the response of the plant to external environmental stress by measuring the maximum quantum efficiency of PS II (Parkhill et al., 2001). The Fv/Fm ratio (as an indirect indicator of stress) typically ranges between 0.81 and 0.83 in healthy plants but falls below this level in plants subjected to stress conditions, because Fo increases and Fm decreases (Song and Hansen, 1994). In our study, the Fv/Fm ratio was within the normal range, averaging 0.82, in the control plants, but averaged 0.7 and 0.75 in plants in the UV-A 370 nm and UV-A 385 nm treatments, respectively, thus lower than the normal range, indicating that the plants were stressed by UV-A LED irradiation. In previous studies, UV-A radiation affected directly decrease in the efficiency of the electron transport system of chloroplasts in wheat seedlings and spinach plants (Joshi et al., 1994; Turcsányi and Vass, 2000). The lower Fv/Fm ratio in the UV-A 370 nm plants suggested that even though the amount of energy irradiated (= 30 W/m^2^) was the same, UV-A at wavelengths of 370 nm, which is a shorter wavelength and contains one quantum more energy than wavelengths of 385 nm, was a stronger plant stressor than was UV-A at 385 nm. This implies that the consideration of peak wavelength of UV-A LEDs is required for the use in horticultural areas such as vertical farms or greenhouses.

### Bioactive Compounds

In general, UV irradiation with strong energy potential may damage not only DNA and proteins but also photosynthetic apparatus, thereby adversely affecting plant growth (Diffey, 1991). However, irradiation using UV with relatively weak energy, such as UV-A, induces a variety of protective response mechanisms in plants and accumulation of low-molecular compounds like antioxidants that act to inhibit oxidative damage (Bharti and Khurana, 1997). Here, we found that kale subjected to two UV-A LEDs (370 and 385 nm) with 30 W/m^2^ for 5 days did not exhibit any morphological damage, suggesting that these UV wavelengths were not strong enough to cause permanent oxidative damage. At the same time, the increases in total phenolic content and antioxidant capacity in plants under these levels of UV-A irradiation (**Figure 3**) indicated that the irradiation treatment was within an appropriate range to stimulate biosynthesis of antioxidant phenolic compounds in kale. At 3 h of UV treatment, the significant increase in total phenolic content and antioxidant capacity in plants subjected to UV-A 370 nm, which has higher energy per photon than does UV-A 385 nm, suggested that UV-A 370 nm was a more effective stimulator despite both having the same energy level (30 W/m^2^). However, as UV treatment continued through 4–5 days, the UV-A 385 nm treatment induced a gradual increase in the production of bioactive compounds (total phenolic content and antioxidant capacity, expressed in mg) per unit fresh weight. This is a very intriguing result, in that it is not a concentrated effect given that the UV-A 385 nm treatment did not inhibit but rather increased kale fresh weight. In other words, irradiation with UV-A 385 nm was beneficial to both plant growth and the production of bioactive compounds. A similar trend was observed in microgreens of basil, beet, and pak choi subjected to UV-A LED light (Brazaitytė et al., 2015). This can be explained by the allocation of additional carbohydrates, which resulted from the enhanced photosynthesis triggered by exposure to UV-A irradiation, toward the production of both biomass and secondary metabolites (Ghasemzadeh et al., 2010). In addition, the activation of cryptochromes (Cry), which are photoreceptors for blue and UV-A lights, by UV-A radiation may stimulate the accumulation of phenolic compounds (Brelsford et al., 2019). However, further studies are needed to understand the interactions between photoreceptors and UV-A radiation in terms of biosynthetic pathways of secondary metabolites (Verdaguer et al., 2017).

Typically two classes of phenolic compounds (hydroxycinnamic acids and flavonoids) are involved in epidermal UV screening in plants (Berli et al., 2010). Our results also showed that these UV‐absorbing compounds were found in kale leaves. The phenolic compounds convert short‐wavelength with high‐energy and destructive radiation into longer wavelength, which is less destructive to the cellular structures of a leaf, including the photosynthetic apparatus such as thylakoids (Bilger et al., 2001). Indeed, flavonoids and hydroxycinnamic acids are known to provide better UV-A waveband protection because absorption maxima of flavonoids and hydroxycinnamic acid were 350–390 and 310–332 nm, respectively (Cerovic et al., 2002; Tattini et al., 2004). The contents of caffeic acid and kaempferol were higher in plants treated with UV-A LEDs at 5 days of treatment, which was a consistent result of total phenolic content and antioxidant capacity. The UV-A LED 385 nm treatment was more effective in enhancing caffeic acid content than was the UV-A 370 nm treatment, indicating that the energy level of UV-A LED 385 nm was more appropriate for increasing production of the individual phenolic compounds, as was observed for total phenol content and antioxidant capacity. However, Moreira-Rodríguez et al. (2017) reported that contents of gallic acid hexoside I (GAH I), gallic acid hexoside II (GAH II), gallotannic acid (GTA), p-hydroxybenzoic acid (p-HBA), and gallic acid derivative (GAD), which are also phenolic compounds, were higher in broccoli sprouts exposed to elevated UV-A levels (4.05 W/m^2^) compared to plants exposed to lower UV-A levels, indicating that the energy level of UV-A is a critical factor for stimulating the increased production of individual phenolic compounds in broccoli sprouts. These contrasting results of the optimum energy level of UV-A imply that even if different plant species are treated with the same UV-A dosage, they may respond in different ways, as for each there is a unique optimal level of UV intensity. Differential response of phenolic compounds according to the level of UV-A and -B radiation was also reported by Heinze et al. (2018). In the study, various kaempferol glycosides and hydroxycinnamic acid derivatives increased in pak choi plants irradiated with UV-B (0.0049 W/m^2^) and UV-A (50 W/m^2^) compared to plants exposed to low UV conditions (UV-B: 0.0006 W/m^2^, UV-A: 1 W/m^2^). Meanwhile, Neugart et al. (2012) suggested that temporary radiation of UV-B affects the accumulation of phenolic compounds such as flavonol glycosides and hydroxycinnamic acid derivatives in kale plants but the pattern was complicated and there was an effect of structure-dependent reaction on the biosynthesis of these phenolic compounds. Given all, each group of phenolic compounds may change depending on the UV energy-dose, chemical structure, and individual plant species (Olsson et al., 1998; Reifenrath and Müller, 2007; Neugart et al., 2012).

Irradiation with UV is known to activate the expression of several genes encoding important enzymes, including PAL, CHS, and F3H, in the phenylpropanoid pathway (Fuglevand et al., 1996). PAL and CHS are key enzymes that catalyze the initial stages of phenylpropanoid and flavonoid biosynthesis, respectively, and F3H is an essential enzyme in the flavonoid biosynthetic pathway, as it catalyzes the 3-hydroxylation of (2S)-flavanones, such as the conversion of naringenin to dihydroflavonols (Wanner et al., 1995; Liu et al., 2013). The activities of these enzymes ultimately lead to the production of UV-absorbing phenolic compounds and thus mitigation of plant-tissue damage caused by exposure to UV light (Middleton and Teramura, 1993). Our results also demonstrated that short-term irradiation with UV-A LEDs activated the transcript level of the PAL, CHS, and F3H genes in kale leaves and increased PAL enzyme activity. PAL gene expression significantly increased in plants exposed to both UV-A 370 nm and UV-A 385 nm after 3 days of treatment and continued to increase after 4 and 5 days. Our results corroborated those found previously for lettuce and tomato, in which PAL gene expression and PAL enzyme activity increased in lettuce and tomato plants exposed to UV-A irradiation for 7 days and 24 h, respectively (Guo and Wang, 2010; Lee et al., 2014). The transcript level of the CHS gene significantly increased after 3 days of treatment, similar to that of the PAL gene, whereas the transcript level of the F3H gene increased but not to statistically significant levels. Likewise, previous research has shown that exposure to UV-A and UV-B activates the CHS and F3H genes in turnip and lettuce, respectively (Zhou et al., 2007; Liu et al., 2013). In addition, low level of UV-A light activated the transcript level of UVR8 gene, which is generally activated by UV-B radiation and promotes the production of antioxidants (Brelsford et al., 2019). In the study, Arabidopsis accumulates flavonoids by stimulating in response to low UV-A dose (peak emission; 365 nm, 15 μmol m^−2^ s^−1^). The response of genes related to secondary metabolite productions to UV-A irradiation is further evidence that the results of the significantly increased total phenol content and antioxidant capacity after 4 and 5 days of UV-A treatment were due to activation of the biosynthetic pathways responsible for the production of secondary metabolites. It has been reported that UV treatment increases flavonoid concentration (Reifenrath and Müller, 2007), UV-absorbing pigments, flavonols, and total antioxidant activity (Csepregi et al., 2017) in leaves of all ages (within a single plant), although there is a difference in the degree of increased. Therefore, the difference in the degree of bioactive compounds increased by UV-A LED treatment is also considered to be negligible, because samples of each parameter were collected from leaves of the same age.

### Reactive Oxygen Species (H_2_O_2_ and O_2_^·−^)

Reactive oxygen species are reactive chemicals with unpaired electrons in the outer orbit and are composed of free radicals and non-free radicals such as hydrogen peroxide (H_2_O_2_), superoxide (O_2_^·−^), singlet oxygen (^1^O_2_), and hydroxyl radical (·OH) (Riley, 1994). ROS generally occur when energy is generated from nutrients and oxygen under normal conditions (Poljsak et al., 2013), and levels of ROS normally rise when a plant is exposed to environmental stress, including exposure to UV light (Couée et al., 2006; Heinze et al., 2018). In our study, contents of O_2_^·−^ and H_2_O_2_ increased in plants treated with UV-A 370 nm compared to control plants at 3 h of treatment, indicating that ROS was rapidly generated in response to UV-A stimulation (**Figure 5**). This rapid increase of ROS, called reactive oxygen burst, is the earliest responses of plant cells under various abiotic stress and regulates signaling network that controls responses downstream (Bhattacharjee, 2005). Razem and Bernards (2003) also reported that initial ROS burst occurred within 1 h of exposure to abiotic stresses such as wounding in carrot plants. Moreover, we observed dynamic changes in ROS levels in the controls as well as in plants in both UV treatments, but UV treatment appeared to alter the pattern of change in ROS levels observed in the control plants. Changes in O_2_^·−^ followed a similar pattern to that of H_2_O_2_, albeit with a ∼1-day delay, which can be explained by the fact that superoxide anion radicals are converted into H_2_O_2_ spontaneously or by superoxide dismutase (SOD) in the reaction pathway (Kyaw et al., 2004).

Manipulation of ROS levels in plants is associated with a positive feedback loop between ROS perception and ROS production (Mittler et al., 2004). In plants, oxidative damage by ROS produced under normal conditions is usually mitigated by enzymatic and non-enzymatic antioxidants, thus ensuring that normal cellular redox homeostasis between the ROS and the antioxidants is maintained, and ROS levels are subsequently kept within a certain range. However, excess ROS are often produced when plants are subjected to environmental stress, which acts as an oxidative stress signal to stimulate the activation of stress-response mechanisms, including antioxidant production (Dixon and Paiva, 1995; Chen et al., 2008; Miller et al., 2010). Jacobo-Velázquez et al. (2011) reported that wounding and hypoxia stresses elevated the level of ROS in carrot plants, which activated the transcript level of PAL resulting in enhancement of total phenolic content. Our results also demonstrated the correlation between ROS generated by UV-A exposure and antioxidant response: because the UV-A 370 nm treatment involved higher energy levels than the UV-A 385 nm treatment, ROS levels rapidly increased after 3 h in plants treated with UV-A 370 nm, following which antioxidant activity also sharply increased in response to the higher contents of ROS. After that point, antioxidant levels began to fall until they reached levels similar to those observed in control plants after 2 days of treatment (**Figure 3B**). The no accumulation of antioxidants in kale irradiated by UV-A LEDs at the initial treatment stage may be explained by excessive ROS production compared to antioxidants that quench ROS. A similar trend was observed in tobacco leaves subjected to UV-B light (Shen et al., 2017). However, antioxidant capacity then increased once more after 3 days of treatment, despite ROS levels staying more or less the same. This may be because the initial 2 days of treatment was a period of adjustment for plants exposed to UV irradiation, and UV-A 370 nm irradiation acted as a mild stress that led to a slight increase in antioxidant phenolics production after 3 days of treatment. In contrast, ROS production was not excessive in plants exposed to UV-A 385 nm because the energy level at this wavelength is considerably lower than at the UV-A 370 nm wavelength, but exposure to UV-A 385 nm may still be sufficient to induce antioxidant synthesis as a stimulatory signal given that antioxidative phenolics production was more efficient in kale plants exposed to UV-A 385 than exposed to UV-A 370 nm. This point was very interesting, but we need further study to clearly elucidate the effect of UV-A wavelength on growth and the accumulation of secondary metabolites in plants.

## Conclusion

Short-term irradiation using UV-A LEDs of different peak wavelengths (370 and 385 nm) improved several growth parameters and enhanced the production of antioxidant phenolic compounds in kale, with effects more pronounced in plants exposed to UV-A 385 nm, which is closer to the visible light range than is UV-A 370 nm. Treatment with UV-A LEDs increased ROS levels in the plant, which stimulated the production of secondary metabolites through overexpression of several key genes (PAL, CHS, and F3H) important to the biosynthetic pathways of these compounds and activation of the PAL enzyme. Thus, contents of total phenolic and individual phenolic compounds, as well as antioxidant capacity, increased in response to UV-A LED irradiation. Our results suggested that short-term exposure to UV-A LED irradiation represents an effective means of stimulating the production of certain secondary metabolites, including phenolic compounds, in kale. However, given that excessive levels of UV irradiation cause damage to plants, additional research focusing on developmental stage, energy dose, and various UV-A peak wavelength is needed to establish the specific optimal standards for crop species and varieties.

## Data Availability

The datasets generated for this study can be found in GenBank, KF218591.1, FJ849059.1, EF531094.1, and EF531096.1.

## Author Contributions

J-HL carried out the measurements and data analysis and drafted the manuscript. K-HS participated in the part of measurements and gene expression analysis. M-MO made a substantial guide about experimental design and critically revised the manuscript.

## Funding

This study was supported by the fund of National Research Foundation of Korea (NRF-2014R1A1A1007793).

## Conflict of Interest Statement

The authors declare that the research was conducted in the absence of any commercial or financial relationships that could be construed as a potential conflict of interest.
